# A Critical Review of Palladium Nanoparticles Decorated in Smart Microgels

**DOI:** 10.3390/polym15173600

**Published:** 2023-08-30

**Authors:** Muhammad Arif

**Affiliations:** Department of Chemistry, School of Science, University of Management and Technology, Lahore 54770, Pakistan; muhammadarif2861@yahoo.com

**Keywords:** Pd nanoparticles, microgels, hybrid microgels, catalysis, classifications, synthesis

## Abstract

Palladium nanoparticles (Pd) combined with smart polymer microgels have attracted significant interest in the past decade. These hybrid materials have unique properties that make them appealing for various applications in biology, environmental remediation, and catalysis. The responsive nature of the microgels in these hybrids holds great promise for a wide range of applications. The literature contains diverse morphologies and architectures of Pd nanoparticle-based hybrid microgels, and the architecture of these hybrids plays a vital role in determining their potential uses. Therefore, specific Pd nanoparticle-based hybrid microgels are designed for specific applications. This report provides an overview of recent advancements in the classification, synthesis, properties, characterization, and uses of Pd nanostructures loaded into microgels. Additionally, the report discusses the latest progress in biomedical, catalytic, environmental, and sensing applications of Pd-based hybrid microgels in a tutorial manner.

## 1. Introduction

Polymers are currently under extensive research for their potential applications across various fields such as packaging [1], aerospace [2], electronics [3], medical devices [4], and automotive [5]. These polymers are classified based on their structure into branched [6], linear [7], and cross-linked [8,9,10] types. The cross-linked polymer network systems that swell in a suitable liquid are known as “gels” [11]. Those gel systems that possess three-dimensional cross-linked networks with micro-scale (diameters) are referred to as “microgels” [12]. In recent years, microgels have gained significant interest in various fields such as nanotechnology [13], biological science [14], and catalysis [15]. The microgels that exhibit a rapid response to external stimuli like pH [16], ionic strength [17], and temperature [18] are called smart microgels, and this property of microgels makes them suitable for a wide range of applications. Moreover, smart polymer microgels are utilized as micro-reactors for the production and stabilization of inorganic nanoparticles, providing extended stability to these nanoparticles [19].

Inorganic nanoparticles find diverse applications across various fields, but their stability requires an appropriate stabilizing system. Polymer microgels have shown the capability to stabilize inorganic nanoparticles over long periods, making them promising candidates for the production and stabilization of inorganic nanoparticles in various ap-plications [20]. The production of noble metal nanoparticles within microgels has been extensively studied, and the loading of nanoparticles into pre-formed polymer microgels has been the subject of intense research. The palladium nanoparticles loaded in smart microgels have more advantages than other noble metal nanoparticle-loaded microgel systems due to their higher catalytic performance and responsive behavior. For instance, palladium nanoparticle-loaded microgel systems showed responsive behavior, which affected their performance in catalysis, biological, and environmental fields. Haleem et al. [21] synthesized the Ag or Pd nanoparticles loaded poly(N-isopropylacrylamide-co-2-acrylamido-2-methylpropane sulfonic acid) P(NIA-AMS) hybrid microgel system. The synthesized system showed swelling and de-swelling behavior under various values of temperature and pH. Pd-based hybrid systems showed better catalytic activity than Ag-based hybrid systems for catalytic reduction reactions of 4-nitrophenol (4NiP) and rhodamine-B (RhAB) under different conditions of temperature and pH of the medium. The swelling and de-swelling behavior also control the release and loading of metal amounts into the microgels. Therefore, these parameters directly affect their antibacterial activity. Dhnavel et al. [22] synthesized Pd nanoparticles in a cross-linked network of chitosan (ChS). They used these hybrid systems for catalytic reduction of 4NiP and for antibacterial activity. The systems showed good results for both gram-positive and gram-negative bacteria. The systems in which more Pd nanoparticles were loaded showed better antibacterial results. Therefore, the Pd-NP-loaded microgel systems can be used in biological fields. The Pd-microgel systems are also applicable for the removal of pollutants from water. Microgels are the most suitable adsorbents, and Pd nanoparticles are the most suitable catalysts. Thus, Pd-microgel systems are the best for the removal of pollutants from water because such systems act as adsorbents as well as catalysts, as reported by Derami and his coworkers [23]. These systems removed positively, negatively, and neutrally charged dyes from water through adsorption as well as catalysis. Additionally, they exhibit superior colloidal stability compared to other gold nanoparticles loaded in microgels. Palladium nanoparticle-based hybrid microgels have gained considerable attention due to their diverse applications in sensing [24], catalysis [25], and biological fields [22]. The responsive nature and environment-dependent characteristics of smart microgels enable their diverse applications in hybrid microgel forms. Microgels maintain their 3D cross-linked networks and the ability to swell or de-swell in a suitable solvent, even with the inclusion of palladium nanoparticles [26]. Palladium-microgel composites possess the properties of both polymeric systems and palladium nanoparticles, making them highly valuable.

Numerous original research articles have been published on palladium-based hybrid microgels over the past decade [27,28,29] but a review article on palladium-based hybrid microgels has not been reported yet. Therefore, it is very important to provide a review to the readers that provides all information related to palladium-based hybrid microgels. This document aims to provide all reported information related to morphologies, synthetic methods, responsive behaviors, and applications of palladium-based hybrid microgels during the previous years. The synthesized morphologies, along with their comparison of potential applications (applied in previous articles or applicable in the future), are discussed in Section 2. The synthetic methods and characterization techniques used for sample analysis of hybrid microgels are explained in Section 3 and Section 4, respectively. In Section 5 and Section 6, the responsive behavior and applications of palladium-based hybrid microgels are explained, respectively. The conclusion and future directions of hybrid microgels are highlighted in the last section.

## 2. Classifications

**Figure 1:**
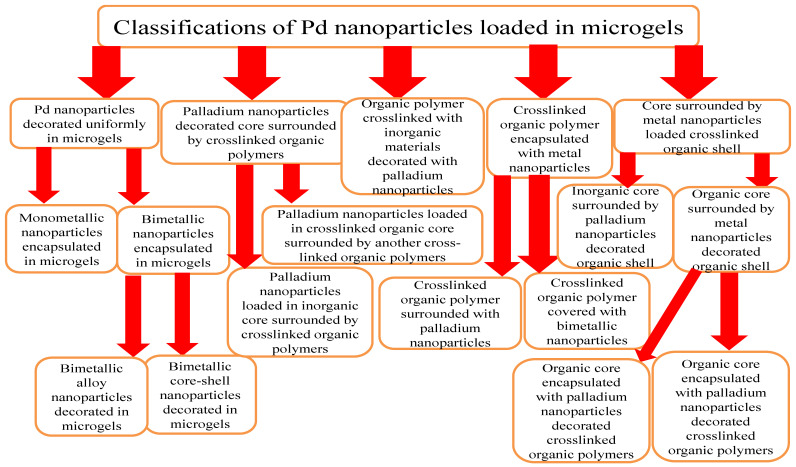
Classification of Pd nanoparticles decorated in smart microgels.

The various classes of palladium-based hybrid microgels can be categorized based on their morphology. Classifications of Pd nanoparticles loaded in microgels are shown in Figure 1. The following sections will elaborate on these classes one by one.

### 2.1. Pd Nanoparticles Decorated Uniformly in Microgels

In such a class, the palladium nanoparticles are uniformly distributed into the cross-linked network of microgels. This class is further divided into two classes, as given below.

#### 2.1.1. Monometallic Nanoparticles Encapsulated in Microgels

In such a class, single metallic (Pd) nanoparticles are present in the cross-linked network of organic polymers. Such a type of class is broadly reported in the literature [30,31,32]. These types of hybrid microgels are very effective for catalysis, adsorption processes, and drug loading because the substrate can easily move inside and outside the crosslinked polymeric network. The performance of such systems can be increased/decreased by varying the conditions of reactions such as pH, temperature, and ionic strength. Chen et al. [33] have synthesized this type of hybrid system and used it as a catalyst. They used it for the catalytic reduction of 4NiP. The catalytic performance of synthesized systems was studied at various temperatures (20–50 °C). The catalytic performance of hybrid microgels decreased with increasing temperature due to de-swelling behavior. Vescovo et al. [34] have also reported the synthesis and catalytic performance of this type of system.

Such types of hybrid systems are mostly used for catalytic reactions. But a detailed study of catalytic performance has not been done yet. The factors directly affecting catalytic performance, such as temperature, pH of the medium, and ionic strength, are not studied broadly. These systems are still not applied for drug delivery due to the toxic nature of Pd. But they can be used as adsorbents.

#### 2.1.2. Bimetallic Nanoparticles Encapsulated in Microgels

In such hybrid systems, bimetallic (palladium along with another metal) nanoparticles are uniformly distributed into the sieves of crosslinked polymer microgels. Such types of bimetallic nanoparticles in microgels are also frequently reported in the literature [35,36]. Such types of systems are further divided into two classes, as given below.

#### 2.1.3. Bimetallic Alloy Nanoparticles Decorated in Microgels

In this type of hybrid microgel, two types of metals (palladium and another metal) are used for the synthesis of metal alloys in nano-sized particles that are present in the crosslinked network of microgels. Such types of hybrid microgels have better catalytic activity than mono-metallic nanoparticles in microgels. The bimetallic systems have a synergistic effect that increases the catalytic performance of hybrid systems. Therefore, bimetallic alloy nanoparticles in microgels are frequently used as catalysts for catalytic reactions. The catalytic performance can also be further increased/decreased by changing the reaction environment. Li et al. [37] have synthesized bimetallic (Fe/Pd) alloy nanoparticles in microgels. They used these systems for the removal of chlorine from trichloroethene (TrCE). They also studied the effect of pH and temperature on the catalytic removal of chlorine. The pH of the medium and temperature conditions directly affected the removal of chlorine from TrCE due to swelling and de-swelling behavior. Nadagouda and Varma [38] have also reported the synthesis of such bimetallic hybrid microgel systems.

Such hybrid systems are applied for the catalytic reduction of only 4NiP. These systems can be applied to the reduction of other nitroarenes. Different types of organic dyes are also reduced/degraded with these bimetallic hybrid systems. These systems are not applied to the adsorption process. They have polar and non-polar parts in their structures. Therefore, these hybrid systems can also be applied for the adsorption of pollutants from water.

#### 2.1.4. Bimetallic Core-Shell Nanoparticles Decorated in Microgels

In such hybrid microgels, the core shell system of bimetallic nanoparticles is present in the crosslinked network of microgels. Such types of hybrid systems are also frequently reported in the literature. The core shell systems of bimetallic nanoparticles have better catalytic activity than monometallic nanoparticles. Therefore, the catalytic activity of bimetallic core shell nanoparticles loaded in microgels shows better catalytic performance than that of monometallic nanoparticles loaded in microgels. Different pollutants can also adsorb on the surface of such hybrid systems. Kakar et al. [39] have reported the synthesis of such types of systems. They found their catalytic performance for the catalytic reduction of 4NiP and methylene blue (MeB). The catalytic performance of bimetallic (Cu/Pd) nanoparticles in microgels was found to be greater than that of monometallic (Cu or Pd) nanoparticles. They also studied the catalytic performance under different conditions of temperature (30–36 °C). The species were rapidly reduced by increasing the temperature, which increases the kinetic energy of reactant molecules (which rapidly reached the surface of metal nanoparticles). Nemygina et al. [40] have also reported bimetallic core-shell (Au = core, Pd = shell) nanoparticles in microgels. They used these hybrid systems as catalysts for Suzuki coupling reactions.

These systems have excellent catalytic performance, but such hybrid systems are not applied for the reduction of broad types of nitroarenes and organic dyes. They can be applied to coupling reactions. Due to the presence of swelling and de-swelling behaviors, they are applicable for drug delivery.

### 2.2. Palladium Nanoparticles Decorated Core Surrounded by Crosslinked Organic Polymers

In such systems, a core shell system of microgels is formed, and palladium nanoparticles are present in the core region of the core shell microgels. The core of core shell microgels can be made with inorganic or organic materials. Therefore, palladium nanoparticles with a decorated core surrounded by crosslinked organic polymer systems are divided into two classes. 

#### 2.2.1. Palladium Nanoparticles Loaded in Inorganic Core Surrounded by Crosslinked Organic Polymers

In this class, the core of core-shell microgel systems is made with inorganic materials, and the shell with organic crosslinked materials. Palladium nanoparticles are present in the inorganic core region of core shell systems. Such systems are very rarely reported in the literature. The density of such systems is very high due to the presence of a solid inorganic core. Therefore, such hybrid microgels can easily be recycled by a simple centrifugation process. Such hybrid systems have lower catalytic activity than uniformly distributed monometallic and bimetallic nanoparticles in microgels. The substrate is not easily reached on the surface of Pd nanoparticles in these systems. More time is required to reach the substrate from the bulk region to the Pd nanoparticles through the crosslinked shell. Therefore, the rate of reduction takes more time as compared to uniformly distributed nanoparticles in microgel systems. Such systems are also more applicable for drug delivery and adsorption processes. Thomas et al. [26] have reported the synthesis of this type of system. They used this system for the reduction of 4NiP. The swelling and de-swelling behavior of hybrid microgels were also studied under various conditions of pH and temperature. This swelling and de-swelling behavior affect the catalytic performance of hybrid microgels.

These hybrid systems are not used for adsorption processes. Due to their stimuli-responsive behavior and polar functional groups, these systems are very effective for the removal of pollutants from water through adsorption. Due to the presence of the aforementioned properties, core-shell hybrid microgels are also applicable for drug delivery. More research can be done on the synthesis and catalytic behavior of such hybrid microgels. Other organic reactions, such as the Suzuki-coupling reaction, can take place in the presence of such hybrid microgels.

#### 2.2.2. Palladium Nanoparticles Loaded in a Crosslinked Organic Core Surrounded by Another Crosslinked Organic Polymer

In such a class, a crosslinked organic core is surrounded by another crosslinked organic polymer, and palladium nanoparticles are present in the core region of core shell microgels. The density of such hybrid systems (organic core and shell as well as organic substance) is lower than that of core and shell microgels (inorganic core and organic shell). These core-shell hybrid systems are made with organic substances. Therefore, their density is low, and they cannot be easily recycled. Such hybrid systems are used for catalytic reactions, but their catalytic activity is less than that of homogenous hybrid microgels. Their catalytic performance can also be increased/decreased by adjusting the pH and temperature of the reaction medium. Sabadasch et al. [41] have reported the synthesis of such hybrid microgels. They used those hybrid microgels as catalysts for the reduction of 4NiP. The pH and temperature affected the hydrodynamic diameter (HDD) of hybrid microgels. So, the reaching capacity of reactant molecules to the surface of Pd nanoparticles can be controlled by adjusting the pH and temperature conditions. Therefore, the catalytic performance of hybrid microgels can be controlled by varying the temperature and pH of the medium. Sabadasch and his coworkers [42] have also reported the synthesis and catalytic activity of this type of hybrid microgel system.

Such types of hybrid microgels are very rarely reported in the literature. More research can be done on such hybrid microgels. The catalytic performance of such systems can also be checked for the catalytic reduction of organic dyes and nitroarenes. Factors such as temperature, pH, and ionic strength can be studied on these hybrid microgels. Different types of pollutants can be removed by these hybrid systems through adsorption.

### 2.3. Core Surrounded by Metal Nanoparticles Loaded Crosslinked Organic Shell

In this class, the organic or inorganic core is surrounded by a crosslinked organic shell, and palladium nanoparticles are present in the shell region of core shell microgels. The core can be made with inorganic or organic materials. Therefore, such classes are further divided into two classes, as given below.

#### 2.3.1. Inorganic Core Surrounded by Palladium Nanoparticles and a Decorated Organic Shell

In this class of hybrid microgels, the core of core-shell microgels is made with inorganic materials, and the shell is made with crosslinked organic polymers. Palladium nanoparticles exist in the shell region of core shell microgels. The density of these hybrid microgels (due to the solid inorganic core) is greater than that of homogenous hybrid microgels with an organic core surrounded by metal nanoparticle-loaded organic shell systems. Therefore, such systems can easily be recycled by simple centrifugation. The catalytic activity of these hybrid microgels is greater than that of the metal nanoparticles present in the core of core shell microgels because the reactant molecules can easily reach the surface of palladium nanoparticles. Such systems can also be used for adsorption and drug delivery. The synthesis of such a type of core-shell hybrid microgel has been reported by Yang et al. [43]. They used these hybrid microgels for Suzuki coupling reactions. The hybrid microgels showed excellent catalytic properties in Suzuki coupling reactions. The catalytic activity of hybrid microgels was affected by changing the solvents. The interaction between the solvent and the shell region of core-shell hybrid microgels is different with different solvents. This interaction affects the swelling behavior of hybrid microgels. Therefore, the solvents affect the catalytic performance of hybrid microgels.

Such types of core-shell hybrid microgels are very rarely reported in the literature. Such types of hybrid systems can be synthesized and used for catalytic reduction of nitroarenes, organic dyes, and Suzuki coupling reactions. Their adsorption process can also be studied deeply.

#### 2.3.2. Organic Core Surrounded by Metal Nanoparticles and Decorated Organic Shell

In this class, the core of core-shell hybrid microgels is made with organic molecules, and the shell is crosslinked with another organic molecule. And the palladium (alone or with another metal) nanoparticles are present in the crosslinked organic polymer shell region of the hybrid core shell system. Such types of core-shell hybrid microgels can be further divided into two classes on the basis of monometallic and bimetallic systems, as given below. 

##### Organic Core Encapsulated with Palladium Nanoparticles and Decorated Crosslinked Organic Polymers

In this class, the core is organic materials, which are surrounded by palladium (alone) nanoparticles loaded with crosslinked organic polymers. Such types of core-shell hybrid microgels are frequently reported. Mostly, the core of core-shell hybrid microgels is made with polystyrene P(SR). In these systems, solid P(SR) is present in the core. Therefore, the density of such core-shell hybrid microgels is also greater. Thus, such systems can also be easily recycled by a simple centrifugation process. These systems can be used for catalytic reactions. The catalytic activity of these hybrid microgels is comparable to that of homogenous hybrid microgels. because the reactant molecules can easily reach the surface of palladium nanoparticles across the crosslinked network. Mei and his coworkers [44] have reported the synthesis of P(SR) surrounded by palladium nanoparticle-loaded crosslinked organic polymers or palladium nanoparticle-loaded linear polymer brushes. They used these hybrid systems for the reduction of 4NiP. The catalytic activity of hybrid brushes was greater than that of core-shell hybrid microgels due to the easier approach of reactant molecules. Such types of classes were reported by Wen et al. [45] and Zhang et al. [46]. They used these systems for Suzuki reactions.

The adsorption process of such core-shell hybrid microgels has not been reported yet. These systems are not used for the catalytic reduction of different nitroarenes, organic dyes, or toxic metal ions. The effects of pH, ionic strength, and temperature have also not been studied yet. These are the available research areas for these hybrid systems.

##### Organic Core Encapsulated with Palladium Nanoparticles and Decorated Crosslinked Organic Polymers

In this class, the core is made with organic materials, and the shell with crosslinked organic polymers, and bimetallic (palladium along with another metal) nanoparticles are present in the shell region of the core-shell system. Such bimetallic core-shell hybrid microgels can be used as catalysts. The catalytic performance of these systems is greater than that of monometallic core-shell hybrid microgels and monometallic homogenous hybrid microgels. The catalytic activity of such classes can be affected by the external environment. Such types of classes are very rarely reported in the literature. Zhang et al. [47] have reported the synthesis of a P(SR) core surrounded by bimetallic (Pd/Ag) nanoparticles loaded with a crosslinked P(NIA) shell. They used these hybrid microgels for the reduction of 4NiP.

Only one system of such a class has been reported in the literature yet. More systems can be designed that are more effective catalysts. The catalytic performance of newly designed hybrid systems can be tested against nitroarenes, organic dyes, and Suzuki reactions. The adsorption property of such systems has also not been reported yet.

### 2.4. Crosslinked Organic Polymer Encapsulated with Metal Nanoparticles

In such a class, the crosslinked organic polymer microgels are surrounded by metal nanoparticles. The metal nanoparticles can be monometallic or bimetallic. Therefore, this class is further divided into two classes on the basis of metal/s nanoparticles, as given below.

#### 2.4.1. Crosslinked Organic Polymer Surrounded by Palladium Nanoparticles

In this class, the crosslinked organic polymer is surrounded by monometallic (palladium) nanoparticles. These types of hybrid microgels are also rarely reported in the literature. They show more catalytic activity than previously discussed hybrid systems due to the direct approach of reactant molecules to the surface of palladium nanoparticles. The reactant takes some time to reach the surface of metal nanoparticles in other systems. But the palladium nanoparticles in these systems are present on the surface of crosslinked polymers. Therefore, no time is wasted during the approach to organic polymers surrounded by palladium nanoparticles. But these systems have the maximum capability for leaching metal nanoparticles during the recycling process. These systems have low adsorption capacity due to covering the surface of crosslinked polymers with nanoparticles. Mutharani et al. [48] have synthesized P(NIA-ChS) microgels covered with palladium nanoparticles. They applied synthesized hybrid microgels for the detection of paraoxon-ethyl (a pesticide).

Newly designed microgels covered with palladium nanoparticle hybrid systems can be synthesized. Their responsive behavior can also be studied in detail. The catalytic performance of such a class of hybrid microgels can be studied in the near future. The factors controlling the leaching of palladium nanoparticles from the surface of microgels are also an area of available research. 

#### 2.4.2. Crosslinked Organic Polymer Covered with Bimetallic Nanoparticles

In these classes, the crosslinked organic polymer is covered with bimetallic (palladium along with another metal) nanoparticles. These hybrid microgels have better catalytic activity than other hybrid systems due to synergistic effects and direct approaches of reactant molecules to the surface of metal nanoparticles. The leaching effect of metal nanoparticles over crosslinked organic polymers during the centrifugation recycling process This is the only drawback of these hybrid microgel systems. These systems can also be used for adsorption processes, but their adsorption capacity is very low as compared to core-shell hybrid microgels and homogenous hybrid microgels. Hu and his coworkers [49] have synthesized crosslinked organic polymers covered with a bimetallic (Au@Pd) core shell system. They applied these hybrid microgels for the catalytic hydrogenation of cyclohexene. They reported that the crosslinked polymers covered with gold nanoparticles did not show catalytic hydrogenation of cyclohexene. The hydrogenation of cyclohexene took place in the presence of bimetallic (Au@Pd) nanoparticles due to a synergistic effect. The microgels surrounded by palladium nanoparticles showed little catalytic activity for hydrogenation.

Only one research article reported such a type of hybrid system. The synthesis of such new hybrid systems is an available topic of research. The catalytic activity of such newly synthesized hybrid systems can be used for the reduction of dyes, nitroarenes, and toxic metal ions. They are also applied for Suzuki coupling reactions. The recycling and stimulus-responsive behavior of such hybrid systems can also be studied in the near future.

### 2.5. Organic Polymer Crosslinked with Inorganic Materials Decorated with Palladium Nanoparticles

In such systems, the organic polymers are crosslinked with inorganic materials, and the palladium nanoparticles are present in the sieves of the crosslinked network. Such hybrid microgels are very rarely reported in the literature. Due to the presence of palladium nanoparticles, these hybrid systems can be used as catalysts. But their catalytic activity is lower than that of microgels covered with monometallic or bimetallic nanoparticles. Nabid and his coworkers [50] have synthesized such types of hybrid microgel systems. They used these systems to oxidize the alcohol. The pH effect of these systems was also studied. The catalytic activity of hybrid microgels increased with the decreasing pH of the medium. At low pH, the nitrogen atoms of pyridines were present in protonated form. Therefore, electrostatic repulsion took place, and the HDD of hybrid microgels increased. Thus, the reactant molecules easily reached the surface of palladium nanoparticles.

All reported morphologies of palladium nanoparticles decorated in microgels are given in Table 1 along with pictorial diagrams. These pictorial diagrams helps to understand the morphology of hybrid microgels.

## 3. Synthesis

Numerous procedures are documented in the literature for synthesizing hybrid micro-gels based on palladium. The method chosen depends on the morphology, design, and architecture of the desired system. The following are different synthetic methods employed for hybrid microgel production.

### 3.1. Formation of Pd Nanoparticles in Already Synthesized Microgels

**Figure 2:**
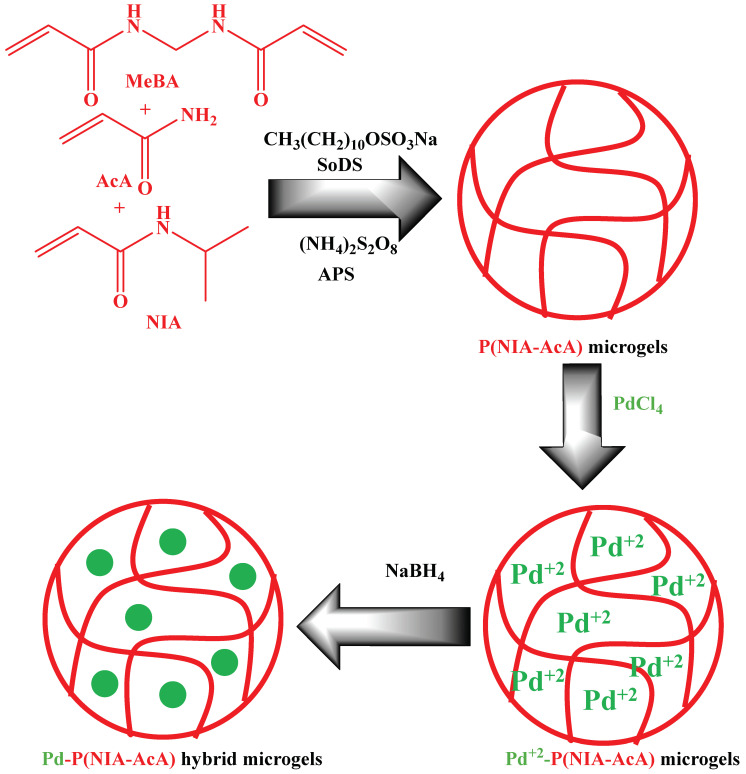
Synthesis of palladium nanoparticles in already synthesized P(NIA-AcA) microgels [30].

In this synthetic method, the synthesis of palladium nanoparticles loaded into microgels consists of two steps. In the first step, the crosslinked polymer microgels are synthesized. These synthesized smart microgels have polar parts in their structures. Therefore, the structure of smart microgels is loaded with palladium ions. After loading the ions, the palladium ions are converted into palladium nanoparticles by the in situ reduction method. Such a type of method is frequently used for the synthesis of palladium nanoparticles in crosslinked polymer microgels. For example, Begum et al. [30] have reported the synthesis of palladium nanoparticles encapsulated in microgels, as shown in Figure 2. They synthesized poly(N-isopropylacrylamide-acrylamide) P(NIA-AcA) microgels in the first step. For this synthesis, NIA, acrylamide (AcA), N, N′-methylenebisacrylamide (MeBA), and sodium dodecyl sulfate (SoDS) were added into a deionized water (95 mL) (DeW) containing a two-neck flask and stirred in nitrogen at 500 rpm at 25 °C. After 30 min, the temperature of the reaction mixture rose to 70 °C and sustained it. Then a small amount of ammonium per sulfate (AmPS) solution was put dropwise into the reaction to start polymerization. The reaction proceeded for a further five h under the same conditions. Palladium nanoparticles were introduced into the sieves of the crosslinked polymeric network of microgels by the in situ reduction method under nitrogen (25 °C). The synthesized P(NIA-AcA) microgel was first diluted, and then the solution of PdCl_4_ was added to the diluted microgel dispersion at 25 °C under N_2_. After 1 h, a NaBH_4_ (freshly prepared) solution was put in to reduce the palladium ions and proceeded for another hour. Similarly, Chen et al. [33] have also synthesized palladium nanoparticles loaded into smart microgels.

### 3.2. Palladium Nanoparticles in Microgels by Mixing Both Palladium Nanoparticles and Microgels

**Figure 3:**
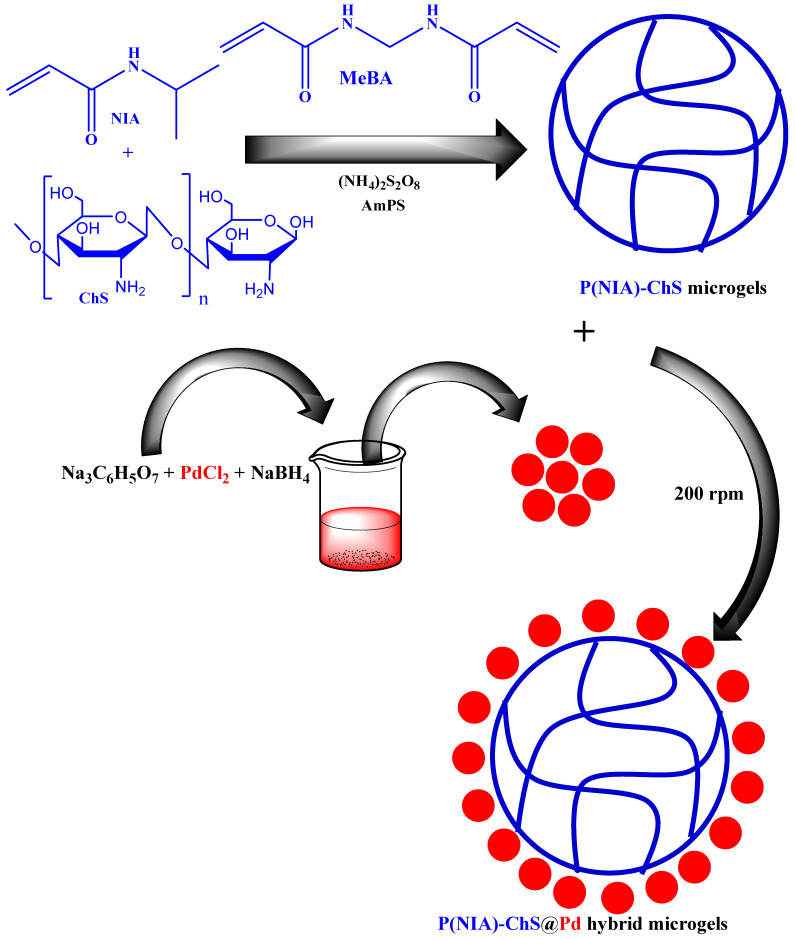
Synthesis of P(NIA)-ChS microgels encapsulated with palladium nanoparticles by mixing palladium nanoparticles and P(NIA)-ChS microgels [48].

In this method, crosslinked polymer microgels and metal nanoparticles are synthesized separately and then mixed to form metal nanoparticles loaded into microgels. The metal nanoparticles have little affinity for the crosslinked network of microgels. That is why metal nanoparticles come onto the surface of crosslinked networks. Such types of synthetic methods are less common than the aforementioned methods. A similar method of synthesis of palladium nanoparticle-loaded microgels was reported by Mutharani et al. [48] as shown in Figure 3. They synthesized poly(N-isopropylacrylamide)-chitosan P(NIA)-ChS microgels. Chitosan (ChS) was dissolved in a small amount of acetic acid and deionized water (DeW) in a beaker and stirred for 24 h. Then the mixture was poured into a three-neck flask and stirred for 25 min under nitrogen. After that, N-isopropylacrylamide (NIA) was added and stirred for 30 min at ambient temperature. Then the temperature increased to 70 °C under N_2_. A small amount of ammonium per sulfate (AmPS) solution was added to the reaction flask to start the polymerization. Then Pd nanoparticles were synthesized separately. For this purpose, trisodium citrate and PdCl_2_ were put into DeW in a separate beaker and stirred for 30 min. NaBH_4_ solution was then added to the beaker to reduce Pd^+2^ ions into atomic form.

These Pd atoms started to coagulate in nano-ranges and were then purified by centrifugation. Palladium nanoparticles were then poured into the dispersion medium of P (NIA)-ChS microgels and stirred for 1 h. Then the palladium nanoparticles were adsorbed on the surface of P(NIA)-ChS microgels to form P(NIA)-ChS@Pd hybrid microgels. Similarly, Liu and his coworkers [51] have synthesized Pd/Ni nanoparticles loaded with acrylamide (AcA) microgels.

## 4. Characterization Techniques

A variety of techniques are utilized to examine the properties and characteristics of palladium nanoparticles loaded in microgels. These techniques also aid in distinguishing smart polymer microgels from their hybrid systems. The following characterization techniques are commonly employed: dynamic light scattering (DLS), wide-angle X-ray scattering (WAXS), ^1^H-nuclear magnetic resonance spectroscopy (NMR), transmission electron microscopy (TEM), scanning electron microscopy (SEM), photo correlation spectroscopy (PSC), UV-Vis spectrometry, Fourier transform infrared spectroscopy (FTIR), X-ray photoelectron spectroscopy (XPS), energy-dispersive X-ray spectroscopy (EDX), differential mechanical analysis (DMA), laser light scattering spectrometry (LLS), differential scanning calorimetry (DSC), atomic force microscopy (AFM), UV/Vis spectrophotometry, attenuated total reflectance spectroscopy (ATR), polarizing optical microscopy (POM), X-ray diffraction (XRD), inductively coupled plasma mass spectrometry (ICP-MS), Brunauer Emmett Teller (BET) [52], inductively coupled plasma atomic emission spectroscopy (ICP-AES), and inductively coupled plasma—optical emission spectrometry (ICP-OES) [53].

Each of these techniques provides specific information about microgels and palladium nanoparticles loaded into smart microgels. Microscopic instruments such as TEM, SEM, and AFM [54] are used to determine the physical appearance of microgel particles and Pd-microgel systems. TEM helps in determining their size and size dispersity, as well as the shape and size of both polymer particles and palladium (alone or with another metal) nanoparticles within the microgels. On the other hand, SEM and AFM [54] investigate the surface morphologies of the organic and inorganic components of hybrid micro-gels.

NMR and FTIR techniques are widely utilized to identify the functionalities of the resulting polymer particles and understand the intermolecular interactions within the polymeric structures and their interaction with inorganic components [35]. XPS, EDX, and XRD methods confirm the metallic nature of the inorganic (palladium alone or with another metal) materials loaded into the polymer microgels [22]. WAXS, DLS, and the Particle Size Analyzer measure the size and diameter of the microgel and hybrid microgel particles. UV/Vis/NIR spectroscopy determines the VPTT (volume phase transition temperature) of the microgel systems and their hybrids and can also verify the fabrication and stabilization of plasmonic nanoparticles within the microgels (in the presence of noble metals along with palladium). TGA, DSC, and DMA are employed to investigate the thermal stability and decomposition of hybrid polymeric networks. TGA can also determine the metal content in microgels loaded with metal nanoparticles [55]. Photon correlation spectroscopy/DLS is used to determine the hydrodynamic size and size distribution of the polymer microgel particles and their hybrids. Wide-angle X-ray diffraction verifies the structure and reproducibility of structures across synthesized hydrogel batches, providing information on the network topology. Rheological measurements help understand the viscoelastic behavior of hydrogel particles. The ICP-MS [23], ICP-AES [56], and ICP-OES [53] techniques were used to determine the amount of palladium nanoparticles in smart microgels. The porosity of microgels and hybrid microgels was determined with BET [52]. This technique showed the surface morphology of hybrid microgels.

The amount of product obtained by catalytic reactions is determined using HPLC and LC-MS techniques [57]. Table 2 summarizes the palladium nanoparticles loaded in polymer microgels with different monomers and co-monomers characterized by these various techniques.

## 5. Properties of Microgels Decorated with Palladium Nanoparticles

**Figure 4:**
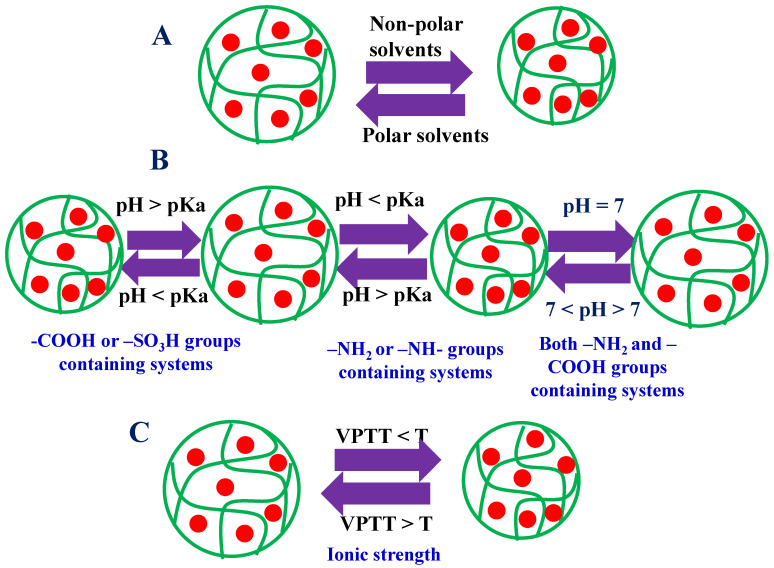
Stimuli-responsive behavior of microgels decorated with palladium nanoparticles (**A**) in polar and non-polar solvents (**B**) at different pHs of the medium with the presence of different functional groups in the structure (**C**) in the presence of ionic strength.

The properties of both microgels and hybrid microgels are directly affected by the environment, such as the pH of the medium, temperature, and solvents, as shown in Figure 4. Let us now discuss these factors. 

### 5.1. pH Effect

Microgels are present in anionic, cationic, and neutral forms. The effect of pH on the swelling and de-swelling behavior of these microgels and hybrid microgels is different, as given below.

#### 5.1.1. Anionic Microgels and Hybrid Microgels

In these types of microgels and hybrid microgels, a negative charge is present in the structure of both microgels and hybrid microgels. Such types of microgels have -COOH or -SO_3_H groups in their structures. These groups are present in protonated form at low pH values (pH ≤ pKa) and deprotonated at high pH values (pH ≥ pKa). The hydrodynamic diameter (HDD) of both microgels and hybrid microgels is greater in deprotonated form than in protonated form. At pH ≥ pKa, the acidic groups (-COOH, -SO_3_H, or both) are presented in a negatively charged form. So electrostatic repulsion takes place due to the presence of the same charges at different places in the structure. Due to this repulsion, the HDD of both microgels and hybrid microgels increases in the protonated form. This electrostatic repulsion depends upon the amount of acidic group containing co-monomers. The swelling and deswelling behaviors are more prominent if the content of acidic groups containing co-monomers is very small. The effect of this behavior gradually decreases with increasing acidic group-containing co-monomers. For example, Sabadasch et al. [65] have reported the synthesis of palladium nanoparticles loaded in poly(N-n-propylacrylamide-methacrylic acid) P(NNA-MeAA) and studied the swelling and de-swelling behavior under various concentrations of MeAA in microgels. They synthesized the microgels using 5 mol% to 25 mol% of MeAA. The microgel synthesized by using 5 mol% of MeAA showed significant swelling and de-swelling behavior, and this swelling and de-swelling behavior gradually decreased by increasing the content of MeAA during the synthesis of microgels. This swelling/de-swelling behavior of hybrid microgels affects their catalytic performance. In a swelling state, the reactant molecules easily reach the surface of metal nanoparticles. Hence, the reaction takes place rapidly. But in the case of de-swelling, the reactant molecules take more time to reach the surface of metal nanoparticles and thus reduce slowly. Dong et al. [66] have synthesized palladium nanoparticles in poly(acrylamide-acrylic acid) Pd-P(AcA-AA). They used this hybrid system for catalytic hydrogenation of allyl alcohols at pH 3 and 8. The catalytic hydrogenation occurred more rapidly at pH = 8 than at pH = 3 due to the swelling state of hybrid microgels in the former case and de-swelling in the latter. Similar behavior of palladium nanoparticles loaded in microgels was reported for the catalytic reduction of 4NiP and RhAB by Haleem et al. [21]. This catalytic performance can be further increased by using bimetallic nanoparticles in microgels due to the synergistic effect. Li et al. [37] have reported bimetallic (Fe/Pd) nanoparticles in microgels. They used these systems for the catalytic removal of chlorine from water.

#### 5.1.2. Cationic Microgels and Hybrid Microgels

Such types of microgels and hybrid microgels have amino groups (-NH_2_, -NH-, -N=CH-) in their structures. These amino groups are pH-sensitive. These groups are present in the protonated form at low pH and in the deprotonated form at high pH. In protonated forms, a positive charge is present on the nitrogen atoms of amino groups. Due to the presence of the same charge in the structure of microgels and hybrid microgels, electrostatic repulsion occurs. Therefore, the HDD of both microgels and hybrid microgels is greater in protonated forms than in de-protonated forms. In this way, the catalytic performance as well as the adsorption capacity of microgels and hybrid microgels can be controlled by swelling and deswelling behavior. Wang et al. [67] have reported the synthesis of palladium nanoparticles loaded in an alginate-polyethyleneimine A-P(EtI) crosslinked organic polymer system. The loading of palladium (II) ions is controlled by adjusting the pH of the medium. These loaded ions were then converted into palladium nanoparticles by in situ reduction. In this way, the crosslinked polymer system also controls the size of metal nanoparticles. The synthesized hybrid system was then used as a catalyst for the reduction of 3-nitrophenol (3NiP). The catalytic performance of those synthesized hybrid systems showed better results in acidic mediums due to swelling behavior. Nabid et al. [50] have reported the synthesis of palladium nanoparticles in Fe_3_O_4_-poly(2-vinyl pyridine) IO-P(2ViP) microgels. They used it for the catalytic oxidation of alcohols under different conditions of pH in the medium. They obtained 96 percent of the product at pH = 5 and 10 percent at pH = 8 under similar conditions. The nitrogen atoms of pyridines were present in the protonated form at pH = 5 and de-protonated at pH = 8. The hybrid microgels were present in a swelling state at low pH, and therefore, a high yield of product was obtained at this pH value. Thomas et al. [26] have also reported similar behavior in another palladium nanoparticle-loaded microgel system for catalytic reduction of 4NiP under different pH conditions.

#### 5.1.3. Both Cationic and Anionic Microgels and Hybrid Microgels

Such types of microgels and hybrid microgels are present in neutral form at pH = 7. Such systems are present in the cationic form at low pH and in the anionic form at high pH. Such types of microgels and hybrid microgels contain both acidic and basic groups in their structures. At low pH, the basic groups are present in protonated form, and repulsion occurs, but at high pH, the acidic groups are present in deprotonated form. Thus, in both conditions, the HDD of microgels and hybrid microgels has a greater value than the neutral form. Such types of microgels and hybrid microgels are very rarely reported in the literature. Mutharani et al. [48] have reported such a hybrid microgel system. They synthesized palladium nanoparticles decorated in poly(N-isopropyleacrylamide)-chitosan P(NIA)-ChS microgels. They used these systems for the detection of paraoxon-ethyl pesticides. They did not study the swelling and de-swelling behavior of these synthesized hybrid systems. More study is required on such types of hybrid microgels.

### 5.2. Temperature Effect

Temperature is also directly affected by the swelling and de-swelling behavior of both microgels and hybrid microgels. The kinetic energy of different parts of the hybrid microgels increases with increasing temperature. This kinetic energy affects the interactions of hybrid microgels. Basically, there are two types of interactions. One force of interaction is hydrophobic, and the other is hydrophilic. Hydrophobic interaction is present between the different parts of hybrid microgels, and hydrophilic interaction is the interaction present between the different parts of hybrid microgels with water molecules. When the temperature increases, the hydrophobic interactions start to dominate the hydrophilic interactions, the HDD of hybrid microgels decreases, and water molecules come out of the sieves of crosslinked networks. On the other hand, when the temperature decreases, hydrophilic interactions dominate over hydrophobic interactions, and hence, more water molecules come into the sieves of the crosslinked network of hybrid microgels. The temperature above which the HDD of hybrid microgels decreases rapidly is called the volume phase transition temperature (VPTT). Above this temperature, the hybrid microgels are in a de-swelling state, and below it, they are in a swelling state. The value of VPTT depends upon the content of polar and non-polar monomers or co-monomers. If the content of the polar co-monomer is high, then the value of VPTT shifts to a higher temperature, and if the content of the polar co-monomer is less, then the value of VPTT is achieved at a low temperature. The value of VPTT also depends on the pH of the medium. Sabadasch et al. [41] have reported the synthesis and swelling/de-swelling behavior of palladium nanoparticles decorated in poly(N-n-propylacrylamide-methacrylic acid)@poly(N-isopropylacrylamide) P(NNA-MeAA)@P(NIA) core shell microgels. They reported the effect of pH on the swelling/de-swelling behavior of core and core shell microgels. At low pH, the carboxylic groups of both core and core shell microgels are present in a protonated form, and hence the interactions are significantly affected by temperature. On the other hand, the carboxylic groups are present in de-protonated form, and the electrostatic repulsion of carboxylate ions minimizes the effect of temperature on hydrophobic and hydrophilic interactions. Chen et al. [33] have also reported the synthesis and swelling/de-swelling behavior of palladium nanoparticles loaded in poly(di(ethylene glycol) methyl ether methacrylate-4-vinylpyridine) P(DEGMEMA-4ViP) microgels. They reported that the hybrid microgels were sifted from a swelling state to a de-swelling state as the temperature of the medium was increased from VPTT. The catalytic activity of hybrid microgels was also decreased by increasing the temperature due to the de-swelling of hybrid microgels. Similar behavior in the temperature of swelling and de-swelling of palladium nanoparticles loaded in microgels was reported by Li et al. [60] and Sabadasch et al. [57].

### 5.3. Nature of Solvent

The swelling and de-swelling behavior of hybrid microgels can be controlled by some suitable solvents. The solvents that have high interactions with the crosslinked network of hybrid microgels can easily come into the sieves of microgels, and hence the HDD of hybrid microgels increases. Those solvents that have low interactions with the crosslinked network of hybrid microgels diffuse difficulty into the sieves of hybrid microgels. Therefore, swelling behavior is also less common in these cases. The solvents, which have a high affinity for the structure of hybrid microgels, diffuse easily along with reactant molecules across the crosslinked polymeric network. Therefore, the catalytic reaction occurs rapidly. Basically, the diffusion of reactant molecules into the sieves of the crosslinked network is controlled by the solvents. The size of molecules in solvents is also controlled by the diffusion of reactant molecules. Therefore, the catalytic activity of palladium nanoparticles in microgels is also controlled by the solvents. Molla et al. [68] have synthesized the palladium nanoparticles loaded in carbazole-based microgels. They used this hybrid system for catalytic N-formylation reactions under various solvents (toluene, THF, dioxane, and dioxane/water). The maximum product yield was obtained in dioxane/water solvent and the minimum in toluene. The dioxane/water solvent has a high affinity for the structure of microgels, and hence a higher yield of product was obtained in this case. Yang et al. [43] have also synthesized palladium nanoparticles in poly(undecylenic acid-co-N-isopropylacrylamide-co-potassium-4-acryloxyoylpyridine-2,6-dicarboxylate)-coated Fe_3_O_4_ IO@P(UNP) microgels. They also studied Suzuki’s coupling reaction under various solvents. More product yields were obtained in the basic medium due to the greater interaction of the base with acidic microgels.

## 6. Applications of Palladium Nanoparticles Decorated in Microgels

**Figure 5:**
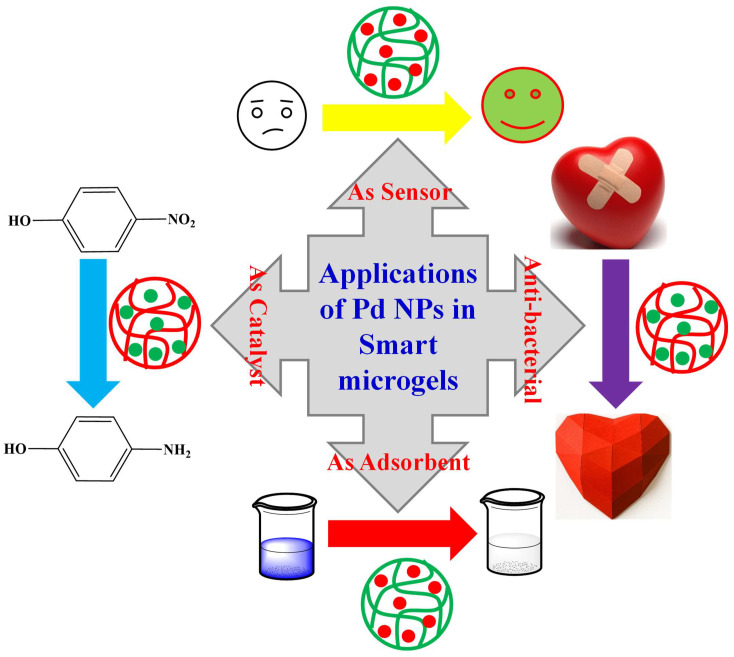
Various applications of palladium nanoparticles encapsulated with smart microgels.

The palladium nanoparticles loaded in microgels have both the properties of microgels and palladium nanoparticles. Therefore, this inorganic-organic combination has various types of applications [22,24,25,69,70] as shown in Figure 5.

### 6.1. As Sensor

The palladium nanoparticles loaded in crosslinked polymer microgels can be used as sensors. This application is mostly monitored with the help of a potentiostat. The sensing property of any material can be determined by cyclic voltammetry. In this technique, the oxidation and reduction positions of the sensor are monitored with cyclic voltammetry. The sensing property is indicated by shifting the oxidation or reduction potential of the sensor in the presence of any substance. A very small amount of any substance can be detected with this technique. Mutharani et al. [48] have reported the use of palladium nanoparticle-loaded microgels for the detection of pesticide (organophosphorus pesticide) paraoxon-ethyl. They used a 0.01 µM to 1.3 mM concentration of pesticide for detection, and excellent results were obtained in this regard. Tang et al. [24] have reported the synthesis of Pd-P (AA). They studied their sensing ability towards H_2_O_2_. They obtained a reduction peak at 0.1 V in the presence of Au/Pd-P(AA) for H_2_O_2_. A similar type of reduction peak was obtained in the presence of Au/Pd^+2^-P(AA), but its intensity is very low. Such a reduction peak was not obtained in the presence of Au/P(AA) for H_2_O_2_.

### 6.2. As Anti-Bacterial

Such systems of hybrid microgels can be used for antibacterial activity. The palladium nanoparticles loaded with microgels have oxidation-reduction potential, as discussed in Section 6.1. Therefore, these hybrid microgels are used for antibacterial purposes. Seku et al. [71] synthesized palladium nanoparticles in microgels and then used them as antibacterials. The synthesized palladium nanoparticles loaded into microgels showed excellent antibacterial activity. Similarly, Dhanavel et al. [22] have synthesized palladium nanoparticles in crosslinked chitosan-based microgels. The hybrid microgels displayed excellent antibacterial effectiveness against both gram-negative and gram-positive bacteria. The minimum inhibitory concentration of the compound against human pathogens was determined in vitro. Additionally, the hybrid microgel was evaluated for its hemolytic activity, and the results demonstrated that the palladium nanoparticles loaded in chitosan-based microgels were non-toxic to red blood cells (RBCs) at concentrations up to 25 μg/mL, as tested.

### 6.3. As Adsorbent

In the structure of hybrid microgels, polar functional groups are present. These polar functional groups of hybrid microgels can interact with polar molecules. Due to this interaction, the polar substance can easily be adsorbed on the surface of hybrid microgels. On the other hand, hybrid microgels that contain non-polar parts in their structures interact with non-polar molecules, and hence non-polar substances can be loaded into such hybrid microgels. During the adsorption process, the molecules of adsorbed materials come to the surface of hybrid microgels from the bulk region due to electrostatic interactions. The concentration of adsorbed material decreases in the bulk region and gradually increases on the surface of hybrid microgels. The adsorption of adsorbent on the surface of the adsorbate can be monitored with a UV-visible spectrophotometer. For example, Mizuno et al. [72] have synthesized and used a palladium nanoparticle-loaded microgel system for the adsorption of 4NiP. 4NiP is a polar compound, and the structure of hybrid microgels also contains polar (-OH) functional groups. Due to this hydrogen bonding, the 4NiP comes into the cavity of the crosslinked structure of hybrid microgels. They monitored this adsorption process with a UV-visible spectrophotometer. Wang et al. [67] have synthesized microgel, which is then used for the sorption of palladium ions and simultaneously for the catalytic reduction of 3NiP. The sorption of palladium ions occurs due to ion-dipole interactions. The loaded ions of palladium were then reduced into palladium nanoparticles and further used for the catalytic reduction of 3NiP.

### 6.4. As a Catalyst

Palladium nanoparticles loaded in microgels can be used as catalysts for various chemical reactions. This catalytic property is due to the presence of palladium nanoparticles in a crosslinked network. Let us now discuss those catalytic reactions one by one.

#### 6.4.1. Catalytic Reduction of Nitroarenes and Organic Dyes

**Figure 6:**
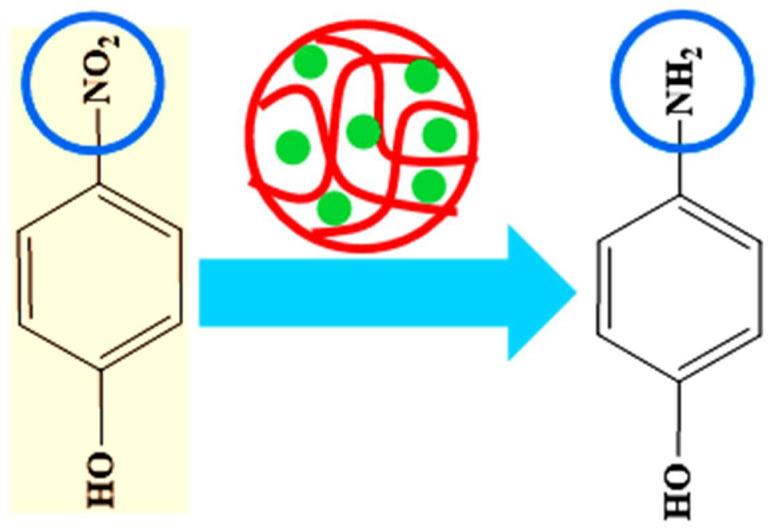
Catalytic reduction of 4NiP to 4AmP in the presence of palladium nanoparticle-loaded microgels [31].

Generally, the palladium nanoparticles loaded in microgels are used as catalysts. The results of the catalytic activity of these systems are excellent. Palladium nanoparticles can easily generate hydrogen from reductants such as NaBH_4_. This generated hydrogen molecule is then used to reduce toxic substances such as nitroarenes and organic dyes. Basically, the palladium nanoparticles in microgels provide the surface for the transformation of hydrogen and electrons from reductant (BH_4_^−^) to oxidant (nitroarenes and organic dyes). Liu et al. [51] have reported the generation of hydrogen from NaBH_4_ by using a palladium nanoparticle-loaded microgel system. After that, Wang and his coworkers [31] synthesized palladium nanoparticles in a crosslinked polymer microgel system and then used them for the catalytic reduction of 4NiP in the presence of NaBH_4_, as shown in Figure 6. Similarly, Derami et al. [23] reported the synthesis of a new hybrid microgel system and then used this system to reduce the 4NiP, MeB, and MeO from water. The catalytic efficiency of palladium nanoparticles loaded in microgels can be enhanced by changing the parameters. Let us discuss all of these one by one.

The catalytic reduction reactions can be enhanced by changing the temperature. Temperature affects the kinetic energy of reactant molecules as well as the interactions present in the structures of hybrid microgels (both hydrophilic and hydrophobic interactions). If the kinetic energy of reactant molecules dominates over the resistance of diffusion rate due to the de-swelling of hybrid microgels, then the reactant reduces rapidly with increasing temperature. And if the resistance of the diffusion rate dominates over the kinetic energy of reactant molecules, then the reduction rate decreases. Chen et al. [33] reported that the reduction rate of 4NiP gradually decreased with increasing temperature. They obtained apparent rate constant values of 2.55 × 10^−3^ min^−1^ at 20 °C and 2.70 × 10^−3^ min^−1^ at 50 °C. In this case, the HDD of hybrid microgels decreased with increasing temperature. Therefore, the diffusion rate of reactant molecules across the crosslinked network of microgels decreased gradually with increasing temperature. Kakar et al. [39] have reported that the value of the apparent rate constant for the reduction of 4NiP and MeB increased with increasing the temperature of the reaction medium. In this case, the kinetic energy of reactant molecules dominates over the resistance of the diffusion rate. Therefore, the reactant molecules reached rapidly on the surface of palladium nanoparticles with increasing temperature and reduced rapidly.

The catalytic efficiency of hybrid microgels can also be changed by changing the pH of the medium. The pH of the medium has an effect on the swelling and de-swelling behavior of hybrid microgels. In the swelling state, the reactant molecules can easily reach the surface of palladium nanoparticles across the crosslinked network of microgels. Therefore, the reduction rate of nitroarenes and organic dyes increases in the swelling state while decreasing in the de-swelling state. Haleem et al. [21] have synthesized sulfonic groups containing hybrid microgels. They reported that the catalytic activity of this hybrid microgel increases with increasing the pH of the medium. The sulfonic groups are converted from protonated form to a deprotonated form by increasing the pH of the medium. So, the HDD of hybrid microgels increases due to electrostatic repulsion. Therefore, the reactant molecules can easily reach the surface of palladium nanoparticles across the crosslinked network. On the other hand, the sulfonic groups are present in the protonated form at low pH values. Therefore, there is no electrostatic repulsion. So, the HDD of hybrid microgels does not increase, and reactant molecules are facing difficulty during diffusion. Similar behavior of palladium nanoparticles loaded in microgels for catalytic reduction of 4NiP was reported by Sabadasch et al. [65]. Similarly, Thomas et al. [26] have reported the catalytic activity of amino groups containing hybrid microgels for the reduction of 4NiP under different pH values. They reported that the catalytic activity of hybrid microgels was found to be greater in an acidic medium than in a basic medium. In an acidic medium, the amino groups are converted into ammonium ions, and electrostatic repulsion takes place due to the same charges. So, the HDD of hybrid microgels increases. In this state, the molecules of 4NiP and BH_4_^−^ diffuse rapidly into the crosslinked network and reach the surface of palladium nanoparticles. Therefore, the reduction rate is high in an acidic medium. On the other hand, the ammonium ions are converted into amino groups in the basic medium. Therefore, there is no electrostatic repulsion. So, the diffusion rate of reactant molecules is less than the diffusion rate in an acidic medium. Therefore, the rate of reduction is higher in acidic media than in basic media.

If the hybrid microgels are used as membranes and reactant molecules such as 4NiP are passed through this membrane in the presence of a reductant (NaBH_4_), The reduction rate of 4NiP can be controlled with pressure. When the pressure of reactant molecules is increased, the reactant molecules rapidly reach the surface of metal nanoparticles across the crosslinked network of microgels. So, the reduction rate of reactant molecules increases gradually by increasing the pressure. Wang et al. [31] have reported similar behavior for the reduction of 4NiP under various conditions of pressure. They reduced the 4NiP by varying the pressure from 0.05 MPa to 0.20 MPa. The value of the apparent rate constant increased from 53 min^−1^ to 113 min^−1^ as the pressure increased from 0.05 MPa to 0.20 MPa. When the pressure increases, reactant molecules (both 4NiP and BH_4_^−^) diffuse more rapidly across the crosslinked polymeric network to the surface of palladium nanoparticles. Therefore, the reduction of 4NiP increases gradually with rising pressure.

The reduction rate of nitroarenes can also be increased by increasing the flow rate of nitroarenes. When the flow rate of reactant molecules goes up, nitroarenes rapidly reach the surface of palladium nanoparticles. So, the reduction rate of nitroarenes rises steadily. Similar behavior has been reported by Wang et al. [31] and Li et al. [60].

#### 6.4.2. Hydrogenation of Different Organic Compounds

**Figure 7:**
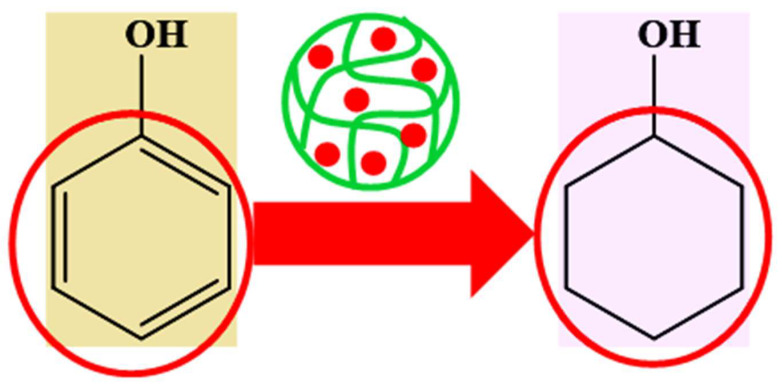
Catalytic hydrogenation of phenol [66].

Hybrid microgels can also be used for the hydrogenation of various organic compounds. Hydrogenation reactions can also be controlled by changing the environment of the reactions due to swelling/de-swelling behavior. Selective hydrogenation can also be performed under specific conditions, mostly using NaBH_4_. Generally, the hydrogenation of unsaturated bonds (double or triple bonds) and carbonyl groups is reported in the literature. Hydrogenation using H_2_ gas is slower than using NaBH_4_. The H_2_ molecule is very stable. Therefore, its reactivity is very low. On the other hand, nascent hydrogen is produced by NaBH_4_. This atomic hydrogen has high reactivity and very low stability. Therefore, it reduces the compound rapidly. Dong et al. [66] have reported the use of palladium nanoparticles loaded in microgels for catalytic hydrogenation of allyl alcohols, as shown in Figure 7. The catalytic activity of those synthesized hybrid microgels showed better catalytic activity in a basic medium than in an acidic medium due to the swelling and de-swelling nature of hybrid microgels. More time was required to complete those hydrogenation reactions (complete in h). Yu et al. [73] have reported the application of synthesized hybrid systems for catalytic hydrogenation of furfural and quinoline. They reported that the catalytic activity of hybrid microgels could be controlled by crosslinking density. If the crosslinking density is high, then the space between sieves is very low, and small palladium nanoparticles are formed in this case. In this case, the surface area of nanoparticles is high. Therefore, the catalytic activity of hybrid microgels is greater in the hypercrosslinked network of hybrid microgels than in the lower-crosslinked network of hybrid microgels. Similarly, Zhang et al. [28] have also reported the hydrogenation of styrene with hybrid microgels and H_2_ gas. Zhang et al. [74] reported the rapid hydrogenation of styrene and other organic compounds in the presence of hybrid microgels and NaBH_4_. They also reported that the hydrogenation of styrene with hybrid microgels in the presence of NaBH_4_ is greater than in the presence of hydrogen gas.

#### 6.4.3. Suzuki Coupling and Heck Reactions

**Figure 8:**
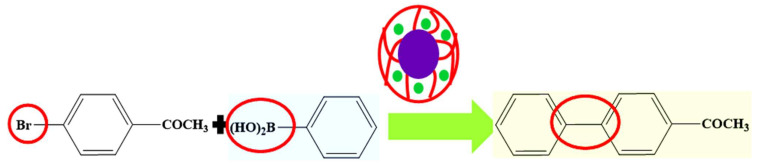
Suzuki-coupling reaction in the presence of palladium nanoparticles in microgels [45].

Palladium nanoparticles decorated in crosslinked organic polymer microgels are frequently used as catalysts in Suzuki coupling and Heck reactions. Homogenous hybrid microgels are mostly reported for such catalytic reactions in the literature [75,76,77]. Some core shell microgels decorated with palladium nanoparticles are also used as catalysts for Suzuki coupling reactions, as reported in the literature [45,46] as shown in Figure 8. Palladium nanoparticles in core shell microgels have an advantage over homogenous systems due to their easy recyclability and remarkable stability. The catalytic efficiency of palladium nanoparticles decorated in microgels can also be further enhanced when another metal, along with palladium, is used for the synthesis of bimetallic nanoparticles decorated in microgels [40]. The catalytic efficiency of hybrid microgels for Suzuki coupling reactions can also be enhanced by adjusting pH [46], temperature [57], and choice of solvent [43].

#### 6.4.4. Some Other Chemical Reactions

**Figure 9:**
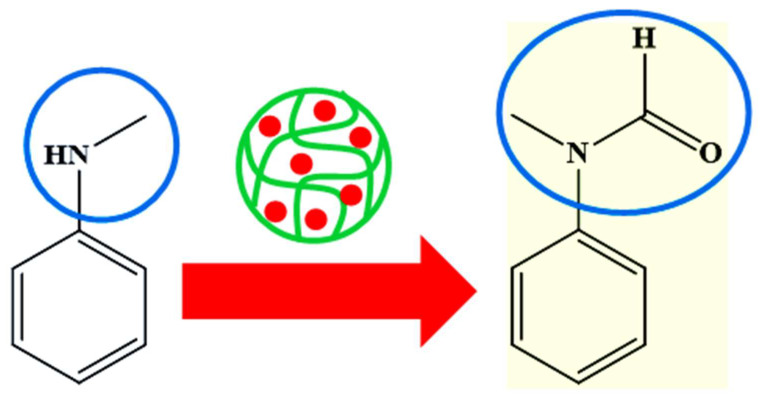
Catalytic N-formylation reaction in the presence of microgels decorated with palladium nanoparticles [68].

Palladium nanoparticles decorated in crosslinked polymer microgels can also be used for catalytic oxidation of alcohols [78], reduction of metal ions [79], N-formylation reactions [68], as shown in Figure 9, degradation of 2-chlorobiphenyl [53], and ester formation [36].

All these applications of palladium nanoparticles encapsulated in crosslinked organic polymers indicate that palladium nanoparticle-containing materials are the best catalysts for all types of catalytic transformation reactions. The catalytic efficiency of these hybrid microgels can be increased in suitable environments.

## 7. Conclusions and Future Directions

The combination of palladium nanoparticles within a crosslinked polymeric matrix produces a fascinating hybrid system that merges the characteristics of organic and inorganic materials. These systems possess electrical and responsive properties, leading to the development of diverse palladium nanoparticle-loaded microgels with distinct shapes, compositions, and architectures, as documented in the scientific literature. Palladium nanoparticles loaded in crosslinked polymer microgels exhibit the best performance for catalytic reactions due to their controlled size and morphology. Therefore, these systems are used mostly for catalytic reactions as compared to other applications. The catalytic performance of hybrid microgels is increased by external stimuli such as pH, temperature, nature of the solvent, and ionic strength. The morphology of hybrid microgels is also affected by their potential application in different fields. The required morphology is obtained by using a suitable synthetic method.

Palladium metal is very toxic to the environment and does not have any potential applications. These metal ions can be converted into some useful materials, such as nanocomposites (palladium nanoparticles decorated with microgels). These composite materials, like palladium nanoparticle-based hybrid microgels, are used in diverse fields. The palladium-based hybrid microgels are not used for drug delivery due to the toxicity of palladium metal. This is an important disadvantage of these hybrid microgel systems. In the realm of smart microgels, palladium nanoparticles have been utilized as catalysts for specific organic reactions, typically for the reduction of 4-nitrophenol. In further research, it would be worthwhile to investigate the potential of palladium nanoparticles within microgels to catalyze diverse organic transformations. Notably, bimetallic nanoparticle-loaded microgels have demonstrated superior catalytic reduction/degradation capabilities compared to microgels based solely on monometallic nanoparticles. Consequently, more extensive examination is required for bimetallic nanoparticle-loaded microgel systems, with palladium as one of the constituent metals. Factors directly influencing the catalytic performance of hybrid microgels, such as the nature of the medium, pH, and ionic strength, warrant investigation in the near future. Furthermore, future research should evaluate the applicability of hybrid polymers by assessing their substrate compatibility.

The application of palladium nanoparticles also increased in the form of hybrid microgels. The polar and non-polar components are present in the structure of the microgel. Therefore, hybrid microgels can be used for catalysis (due to palladium nanoparticles) and adsorption processes (due to microgels). A few palladium nanoparticle-loaded microgel systems have been employed for adsorption processes; other systems should be explored for the removal of additional water pollutants such as heavy metal ions and toxic organic dyes. These hybrid systems hold the potential for purifying industrial waste through both adsorption and catalytic reactions. However, the use of non-biodegradable microgels presents a significant challenge in this application, which can be overcome by incorporating biodegradable monomers during the synthesis of microgels.

## Figures and Tables

**Table 1:** Morphology-based classifications of palladium nanoparticles decorated in microgels along with pictorial diagrams (color indications: Palladium nanoparticles = green color, other metal nanoparticles = blue color, red and yellow colors = organic polymers, and purple color = inorganic particles (SiO_2_ or Fe_2_O_3_/Fe_3_O_4_).

Pictorial Diagram	Morphology of Hybrid Microgels	Abbreviations of Hybrid Microgels	Class of Pd NPs in Smart Microgels	References
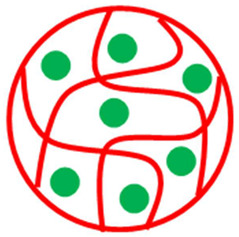	Homogenous microsphere	Pd-P(NIA-AcA)	Pd NPs in P(NIA-AcA)	[32]
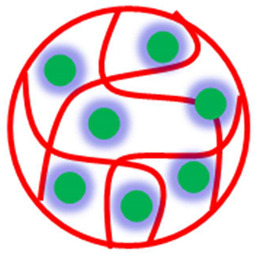	Homogenous microsphere	Fe/Pd-P(AA)	Fe/Pd NPs in P(AA)	[39]
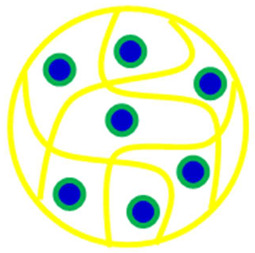	Homogenous microsphere	Au@Pd-P(SR)	Au@Pd NPs in P(SR)	[42]
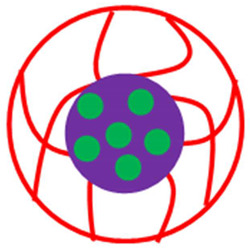	Core shell system (Core = Pd-SiO_2_ Shell = P(DMM))	Pd-SiO_2_@P(DMM)	Pd NPs in SiO_2_ encapsulated with P(DMM)	[28]
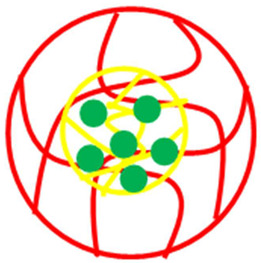	Core shell system (Core = Pd-P(NIA-MeAA Shell = P(NIA-HyMBP))	Pd-P(NIA-MeAA)@P(NIA-HyMBP)	Pd NPs in P(NIA-MeAA) encapsulated with P(NIA-HyMBP)	[44]
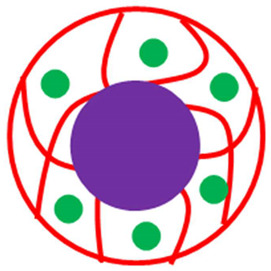	Core Shell system (Core = Fe_3_O_4_ Shell = Pd-P(UNP))	Fe_3_O_4_@Pd-P(UNP)	Fe_3_O_4_ encapsulated with Pd NPs decorated in P(UNP)	[45]
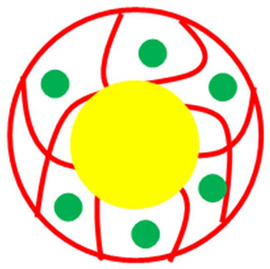	Core shell system (Core = P(SR) Shell = Pd-P(NIA))	P(SR)@Pd-P(NIA)	P(SR) encapsulated with Pd NPs decorated in P(NIA)	[46]
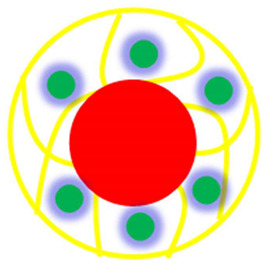	Core shell system (Core = P(SR) Shell = Ag/Pd-P(NIA)	P(SR)@Ag/Pd-P(NIA)	P(SR) encapsulated with Ag/Pd NPs decorated in P(NIA)	[49]
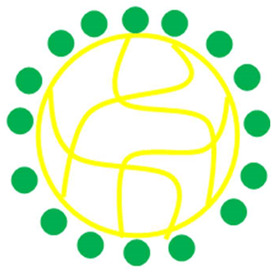	Core shell system (Core = P(NIA-ChS)@Pd NPs)	P(NIA-ChS)@Pd	P(NIA-ChS) encapsulate with Pd NPs	[50]
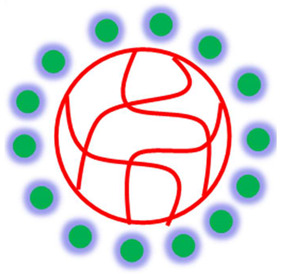	Core shell system (Core = P(ViBIC-IL) Shell = Pd@Au)	P(ViBIC-IL)@(Pd@Au)	P(ViBIC-IL encapsulate with Pd NPs surrounded Au NPs	[51]
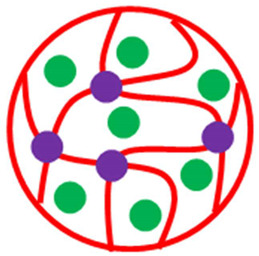	Homogenous microsphere with inorganic crosslinker	Pd-P(ViP)	P(ViP) decorated with Pd NPs (crosslinker = Fe_3_O_4_)	[52]

**Table 2:** Different techniques were used for the characterization of palladium nanoparticles in smart microgels along with metal nanoparticles.

System	Monomers	Metal Nanoparticles	Characterization Techniques	References
Pd-BaNC-P(DA)	DA	Pd	SEM, XPS, TEM, XRD, TGA, UV-Vis, ICP-MS	[23]
Pd-P(MeAA)@HyMBP	MeAA, HyMBP	Pd	UV-Vis, SEM, AFM, AAS	[54]
Pd-P(DiVB-DCIIL)	DiVB, DCIIL	Pd	TEM, XRD, XPS, TGA	[55]
Pd-GO/P(ImZ/ImZI)	GO, ImZ, ImZI	Pd	FTIR, NMR, TGA, TEM, SEM, XRD, UV-Vis, AAS, ICP-AES	[56]
Pd-ChS-GA-RGO	ChS, GA, RGO	Pd	SEM, XRD, TEM, TGA, BET, UV-Vis	[52]
Pd-P(EtI), Ag-P(EtI)	EtI	Pd or Ag	SEM, TEM, XRD, XPS, TGA, BET	[58]
Pd-Ti_3_C_2_-P(DoA)	DoA, Ti_3_C_2_	Pd	XRD, FT-IR, TGA, SEM, TEM, BET, and XPS	[59]
Au/Pd-CeL-CD, Ag/Pd-CeL-CD	CeL, CD	Au and Pd, Ag and Pd	TEM, EDS, SEM, XRD, XPS, NMR	[35]
Pd-ChS	ChS	Pd	XRD, HR-TEM, FTIR, FE-SEM, EDX, UV-Vis	[22]
Pd-P(NIA-AA)	NIA, AA	Pd	FE-SSEM, AFM, XRD, FTIR, ICP-AES	[60]
P(SR)@Ag/Pd-P(NIA)	SR, NIA	Ag and Pd	ICP-AES, DLS, TEM, UV-Vis., SAXS	[47]
Pd@Cu-P(NIA-AA)	NIA, AA	Pd and Cu	XPS, TGA, UV-Vis, XRD, TEM, SEM	[39]
Pd/Ni-P(AcA)	AcA	Pd and Ni	XRD, TEM, ICP-AES, FTIR	[51]
Fe@Pd-P(NIA-MMA)	NIA, MMA	Pd and Fe	ICP-OES, FIB-SEM, ED-XRD	[53]
Pd-P(MMA-4ViP)	MMA, 4ViP	Pd	XRD, TGA/DSC, TEM, XPS, EDX	[34]
Pd-P(NIA-MeAA)	NIA, MeAA	Pd	PCS, HP-LC, LC-MS, NMR	[57]
Pd-P(NIA-AcA)	NIA, AcA	Pd	STEM, EDX, TEM, XRD, TLC, HR-TEM, NMR, UV-Vis	[30]
Cu/Pd-P(ViP), Pt/Pd-P(ViP), Pd/Fe-P(ViP)	ViP	Cu and Pd, Pt and Pd, Pd, and Fe	SEM, FTIR, TEM, UV-Vis, Digital Camera	[38]
Pd-P(AmAm)	AmAm	Pd	UV-Vis, TEM, HR-TEM, FTIR	[61]
Fe/Pd-P(AA)	AA	Fe and Pd	ATR-FTIR, FE-SEM, EDS, XPS, ICP-AES, BET	[37]
Pd-ChS@MnFe_2_O_4_	ChS	Pd, Mn, Fe_2_O_4_	FTIR, TEM, FE-SEM, XPS, XRD, ICP-MS, UV-Vis	[62]
Pd-CD-P(ViDF)	CD, ViDF	Pd	SEM, EDS, TEM, ATR-FTIR, XPS, AFM	[63]
Pd-P(AcA-DMMB-IL)	AcA, DMMB, IL	Pd	FTIR, NMR, UV-Vis, TEM, SEM, EDS, XRD,	[64]

## Data Availability

Data will be provided on request.

## Abbreviations

NIA	N-isopropylacrylamide
3NiP	3-Nitrophenol
4NiP	4-Nitrophenol
AA	Acrylic acid
AMS	2-Acrylamido-2-methylpropane
RhAB	Rhodamine-B
ChS	Chitosan
HDD	Hydrodynamic diameter
DLS	Dynamic light scattering
SoDS	Sodium dodecyl sulfate
EDX	Energy dispersive X-rays
FTIR	Fourier Transformed Infrared Spectroscopy
P(SR)	Polystyrene
LCST	lower critical solution temperature
MeBA	N,N′-Methylene-bisacrylamide
AmPS	Ammonium persulfate
AcA	Acrylamide
SEM	Scanning electron microscopy
TGA	Thermo gravimetric analysis
UV-Vis	UV/Visible spectroscopy
VPTT	Volume phase transition temperature
XPS	X-ray photoelectron spectroscopy
XRD	X-ray diffraction
4ViP	4-vinylpyridine
DeW	Deionized water
A-P(EtI)	Alginate-poly(ethyleneimine)
IO-P(2ViP)	Fe_3_O_4_-poly(2-vinylpyridine)
MeAA	Methacrylic acid
NNA	N-n-propylacrylamide
P(DA)	Polydopamine
BaNC	Bacterial nanocellulose
HyMBP	2-Hydroxy-4-(methacryloyloxy)-benzophenone
P(DiVB-DCIIL)	Poly(divinylbenzene-co-dication imidazole ionic liquid)
GO	Graphene Oxide
P(ImZ/ImZM)	Poly(Imidazol/Imidazolium)
GA	Glutaraldehyde
CeL	Cellulose
CyD	Β-Cyclodextrin
DMMB	Dimethylaminoethylmethacrylate bromododecane
P(UNP)	Poly(undecylenic acid-co-N-isopropylacrylamide-co-potassium-4-acryloxyoylpyridine-2,6-dicarboxylate)
ViBIC	1-vinyl-3-butylimidazolium chloride
